# In vitro evidence for the involvement of H_2_S pathway in the effect of clodronate during inflammatory response

**DOI:** 10.1038/s41598-021-94228-y

**Published:** 2021-07-20

**Authors:** Rosangela Montanaro, Alessio D’Addona, Andrea Izzo, Carlo Ruosi, Vincenzo Brancaleone

**Affiliations:** 1 Department of Science, University of Basilicata, Via Ateneo Lucano, 85100 Potenza, Italy; 2 Humanitas Clinical and Research Center-IRCCS, Via Alessandro Manzoni 56, 20089 Rozzano, Italy; 3 Department of Public Health, Section of Orthopaedics and Trauma Surgery, AOU Federico II, School of Medicine and Surgery, Federico II” of Naples, Naples, Italy

**Keywords:** Molecular biology, Molecular medicine, Rheumatology

## Abstract

Clodronate is a bisphosphonate agent commonly used as anti-osteoporotic drug. Throughout its use, additional anti-inflammatory and analgesic properties have been reported, although the benefits described in the literature could not solely relate to their inhibition of bone resorption. Thus, the purpose of our in vitro study is to investigate whether there are underlying mechanisms explaining the anti-inflammatory effect of clodronate and possibly involving hydrogen sulphide (H_2_S). Immortalised fibroblast-like synoviocyte cells (K4IM) were cultured and treated with clodronate in presence of TNF-α. Clodronate significantly modulated iNOS expression elicited by TNF-α. Inflammatory markers induced by TNF-α, including IL-1, IL-6, MCP-1 and RANTES, were also suppressed following administration of clodronate. Furthermore, the reduction in enzymatic biosynthesis of CSE-derived H_2_S, together with the reduction in CSE expression associated with TNF-α treatment, was reverted by clodronate, thus rescuing endogenous H_2_S pathway activity. Clodronate displays antinflammatory properties through the modulation of H_2_S pathway and cytokines levels, thus assuring the control of the inflammatory state. Although further investigation is needed to stress out how clodronate exerts its control on H_2_S pathway, here we showed for the first the involvement of H_2_S in the additive beneficial effects observed following clodronate therapy.

## Introduction

Bisphosphonates (BPs) are synthetic, non-hydrolysable pyrophosphate analogues containing a P–C–P core and R1 and R2 chains, bound to the central carbon^1,2^. Currently, BPs represent the most widely used treatment for common skeletal disorders mediated by osteoclastic action such as osteoporosis, metastatic bone and Paget's disease, although their exact molecular mechanisms of action has only been clarified in the last decade^1,3^. In particular, BPs mainly inhibit bone resorption, which underlies osteoporosis, together with other pathological conditions such as hypercalcemic malignancies, metastatic osteolytic bone or hyperparathyroidism^4,5^.

BPs can be divided in two main groups, non-nitrogen BPs (NN-BP) such as etidronate, clodronate and tildronate or nitrogen BPs (N-BP) such as pamidronate, alendronate, incadronate, and ibandronate^1,6,7^. Generally, the anti-resorptive potency exhibited by N-BPs is greater than that shown by NN-BPs. This effect has been ascribed to the different mechanism of action^2^.

Clodronate (Clo) is one of the most known NN-BP, in which the two lateral chains are made up of two chlorine atoms^2,7,8^. It has been widely used since the 60 s and today is still effective in the treatment of several osteometabolic disorders featured by excessive bone resorption^8,9^.

Although Clo is commonly used as anti-osteoporotic drug, recently many evidences have shown that Clo has also been successfully tested in erosive osteoarthritis (OA) upon intra-articular and parenteral administration^8,10,11^. In this setting, Clo has been demonstrated to regulate the biosynthesis and the release of NO, expression of cycloxygenase 2 (COX_2_) and pro-inflammatory cytokines such as TNF-α, IL-1β that are known to promote cartilage erosion, subchondral bone alterations and to inhibit progenitors maturation into chondrocytes in OA early stages^7,12–15^. Furthermore, given its additional anti-inflammatory and analgesic properties, Clo is used to treat several musculoskeletal pathologic onsets ranging from complex regional pain syndrome (CRPS) and bone marrow lesions (BMLs) to osteomyelitis, rheumatoid arthritis (RA), hand erosive osteoarthritis and hip pain unresponsive to other drugs^9,11,16–19^. Clodronate is also indicated in patients undergone to prosthetic surgery, starting from the pre-operative stage to the immediate post-surgery to improve prosthesis integration to the bone, or in the treatment of osteolysis in case of aseptic loosening due to wear debris disease^11^.

Therapeutic effect of clodronate appears so various and peculiar as, in many cases, the benefits described in the literature could not be explained by the improvement of symptoms associated exclusively to the inhibition of bone resorption. These features associated to an evidence-based medicine have been disregarded for a long time and there is a lack of studies focusing on additional pathways possibly triggered by clodronate. Indeed, it is feasible that, beyond its known mechanism, other mediators regulated by BPs could play a role, particularly in anti-inflammatory/analgesic effects, nonetheless no evidence so far have unveiled underlying mechanisms.

Inflammation is a protective process that could become detrimental if not adequately controlled. In particular, one of the key mediators in inflammation is hydrogen sulfide (H_2_S), a gaseous molecule endogenously produced in a variety of mammalian tissues mainly from the aminoacids cysteine and homocysteine. Two main enzymes, cystathionine-γ-lyase (CSE) and cystathionine-β-synthase (CBS), dependent on pyridoxal-5-phosphate, produces H_2_S^20^. In addition, H_2_S is also synthesized through a different pathway involving cysteine aminotransferase (CAT) and 3-mercaptopyruvate sulfur-transferase (3-MPST)^21^. Physiological levels of H_2_S ranges normally between 23 and 45 µmol/l, in rodent as well as human serum, although they can vary depending by pathological onsets, such as sepsis or diabetes^22^. H_2_S plays a crucial role during inflammatory processes. Indeed, when inflammation spreads, its release is inhibited. On the contrary, the increase of H_2_S levels assures the resolution of inflammation^23^. Furthermore, H_2_S has been demonstrated to be significantly higher in the synovial fluid than in blood during joint inflammatory onset (Whiteman et al.). However, this behavior may not be specific to RA as other inflammatory diseases, such as psoriatic and reactive arthritis. In fact, significantly higher levels of H_2_S have been found within the synovia fluid in osteoarthritic (OA) patients^24^.

Indeed, the importance of H_2_S in the control of inflammation, the clinically relevant amelioration of inflammatory parameters associated to clodronate, together with the lack of preclinical studies identifying possible additional targets for clodronate, led us to investigate whether H_2_S/CSE pathway could be part of a possible molecular mechanism triggered by clodronate.

## Results

### Effect of clodronate on iNOS and COX_2_ expression

**Figure 1:**
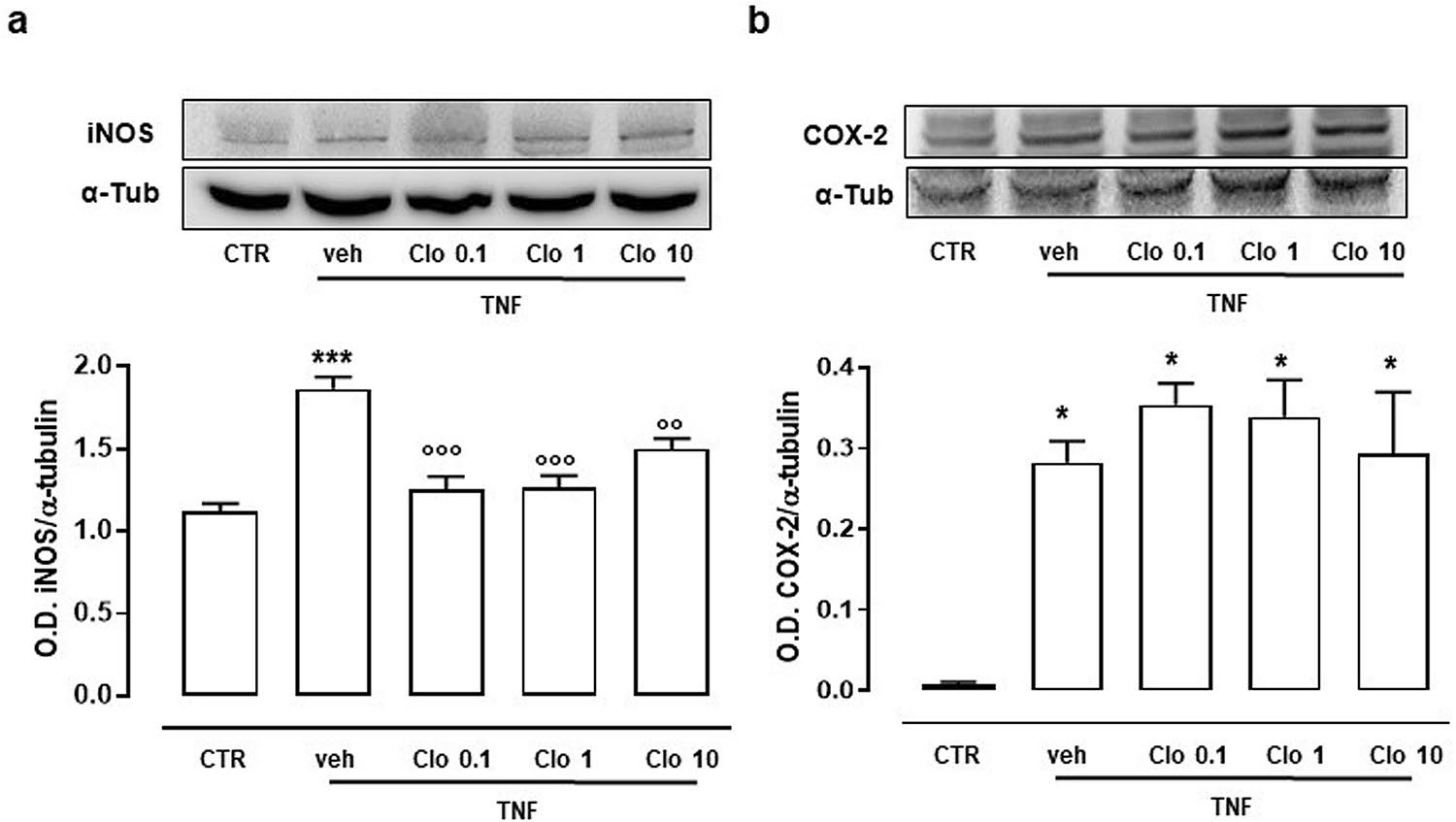
Expression of inflammatory protein levels in FLS stimulated with TNF-α. (**a**) Western blots cropped images for iNOS expression following TNF-α treatment and the effect of clodronate (10 µM) administration. (**b**) Western blots cropped images for COX2 expression following TNF-α treatment and the effect of clodronate (10 µM) administration. Optical density (OD) values have been standardized towards housekeeping protein α-tubulin (α-tub). *p < 0.05 and ***p < 0.001 vs CTR; °°*p* < 0.01 and °°°*p* < 0.001 vs veh. Full-length blots are presented in Supplementary Fig. 2.

In the first set of experiments, FLS have been cultured in presence of TNF-α for 6 h and expression of inducible nitric oxide synthase (iNOS) and COX2 determined. Both iNOS and COX2 expression were elevated by TNF-α treatment, as normally occurring during inflammatory process. The administration of Clodronate, in presence of TNF-α, significantly modulated the iNOS expression (Fig. 1a). Clodronate was already efficacious at the concentration of 0.1 µM. Conversely, clodronate did not affect the expression of COX_2_ (Fig. 1b).

### Effect of clodronate on cytokines expression in FLS stimulated with TNF-α

**Figure 2:**
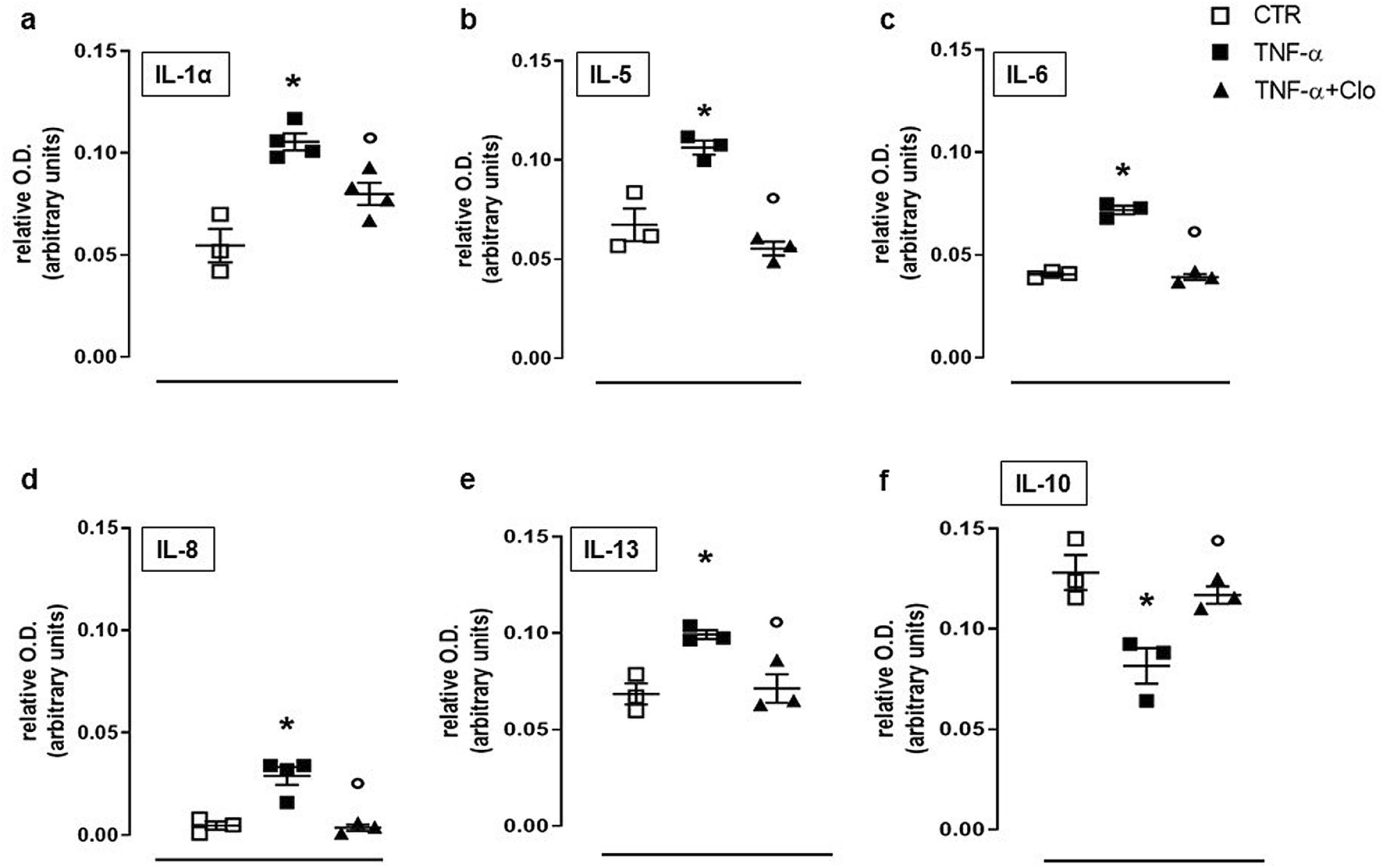
Interleukin levels following TNF-α stimulation in FLS. Expression of IL-1α, IL-5, IL-6, IL-8, IL-13 and IL-10 (**a**–**f**) in untreated FLS (CTR) and following administration of TNF-α + clodronate (10 µM) or TNF-α alone. Data are expressed as relative optical density (OD). *p < 0.05 vs CTR; °*p* < 0.05 vs TNF-α.

To evaluate a possible intrinsic anti-inflammatory activity exerted by clodronate, we further investigated its effects by performing cytokine array to detect changes in expression of 23 different cytokines. Homogenised samples derived from FLS treated with TNF-α, revealed an increase of different pro-inflammatory interleukins levels, such as IL-1, IL-5, IL-6, IL-8 and IL-13. Conversely, the expression of the anti-inflammatory cytokine IL-10 was suppressed by TNF-α treatment (Fig. 2). Administration of clodronate (10 µM) to FLS in presence of TNF-α significantly inhibited cytokine levels elicited by TNF-α. In particular, clodronate significantly reduced IL-1, IL-5, IL-6, IL-8 and IL-13 (Fig. 2°–e). Similarly, clodronate administration brought IL-10 expression back to control level (Fig. 2f).

**Figure 3:**
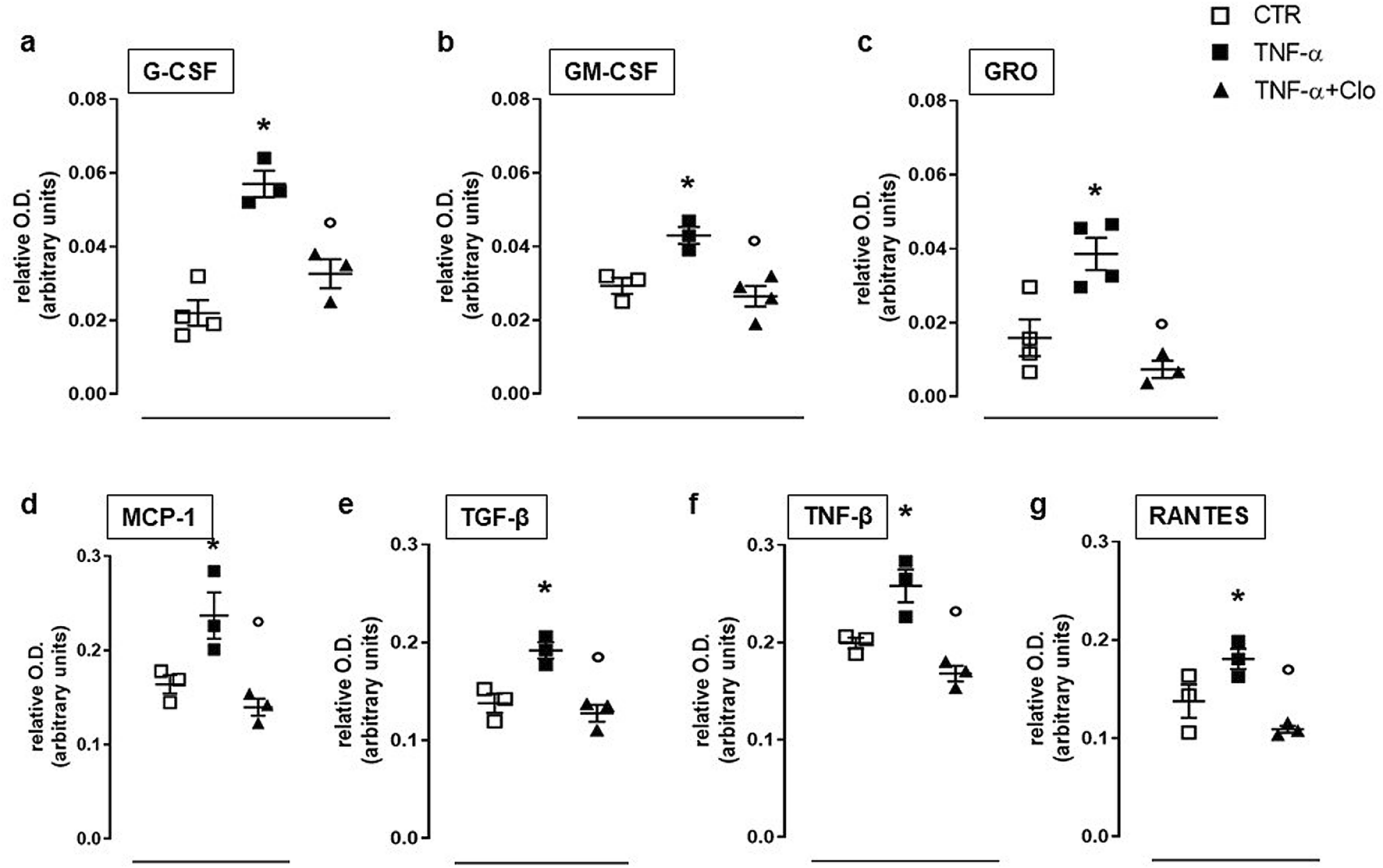
Chemokines levels following TNF-α stimulation in FLS. GCSF, GMCSF, GRO, MCP1, TGF-β1, TNF-β and RANTES (**a**–**g**) expression in untreated FLS (CTR) and following administration of TNF-α + clodronate (10 µM) or TNF-α alone. Data are expressed as relative optical density (OD). *p < 0.05 vs CTR; °*p* < 0.05 vs TNF-α.

TNF-α treatment also increased expression of granulocyte colony-stimulating factor (GCSF), granulocyte–macrophage colony-stimulating factor (GMCSF) and growth-related oncogene (GRO) as well as monocyte chemoattractant protein 1 (MCP1), regulated on activation, normal T cell expressed and secreted (RANTES), Transforming growth factor beta (TGFβ1) and tumor necrosis factor beta (TNF-β) (Fig. 3). Administration of clodronate (10 µM) to FLS in presence of TNF-α significantly suppressed the increased expression of growth factors namely GCSF, GMCSF and GRO elicited by TNF-α (Fig. 3a–c). In addition, relevant MCP1, RANTES and TGF-β1 were similarly reduced to physiological levels following administration of clodronate (Fig. 3d–g).

### Involvement of H_2_S in clodronate effect

**Figure 4:**
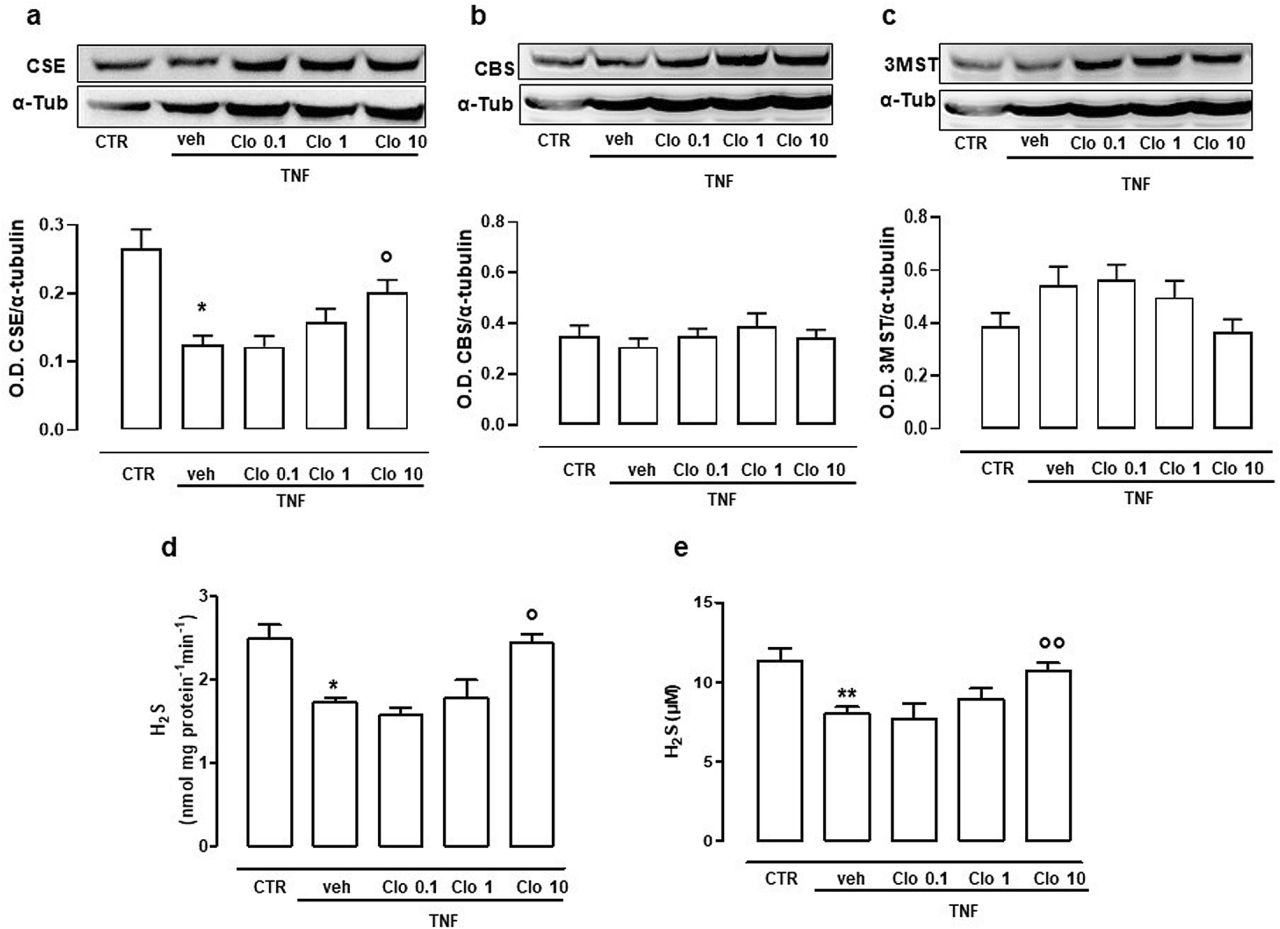
Modulation of H_2_S-synthesizing pathway by clodronate following TNF-α stimulation in FLS. Western blots cropped images of CSE (**a**), CBS (**b**) and 3MST (**c**) expression following TNF-α treatment alone (veh) and with increasing concentration of clodronate (0.1-10 µM). Optical density (OD) values have been standardized towards housekeeping protein α-tubulin (α-tub). *p < 0.05 vs CTR; °*p* < 0.05 vs veh. (**d)** Intracellular H_2_S biosynthesis following treatment with TNF-α alone or in combination with clodronate (0.1–10 µM). Data are expressed as nmol per mg of total protein per minute. *p < 0.05 vs CTR; °*p* < 0.05 vs veh. (**e**) Extracellular H_2_S determination in supernatants from FLS treated with TNF-α alone or in combination with clodronate (0.1–10 µM). Data are expressed as concentration detected (µM). **p < 0.01 vs CTR; °°*p* < 0.01 vs veh. Full-length blots are presented in Supplementary Fig. 3.

In another set of experiments, we measured levels of H_2_S and expression of CSE, CBS and 3MST following TNF-α treatment. Only CSE expression was reduced significantly reduced by TNF-α (Fig. 4a), while neither CBS nor 3MST displayed significant changes (Fig. 4b–c). Conversely, administration of 10 µM clodronate partly restored CSE towards levels similar to those observed in unstimulated cells (Fig. 4a), with little or no effect for CBS and 3MST.

In addition, we evaluated the amount of H_2_S produced in FLS in presence of TNF-α alone or in combination with clodronate. Enzymatic biosynthesis of intracellular H_2_S by CSE was significantly suppressed by TNF-α, however, clodronate reversed this effect (Fig. 4d). Similarly, the levels of H_2_S in supernatant from TNF-α treated cells were reduced and clodronate was able to revert this effect, restoring H_2_S levels to control values (Fig. 4e).

**Figure 5:**
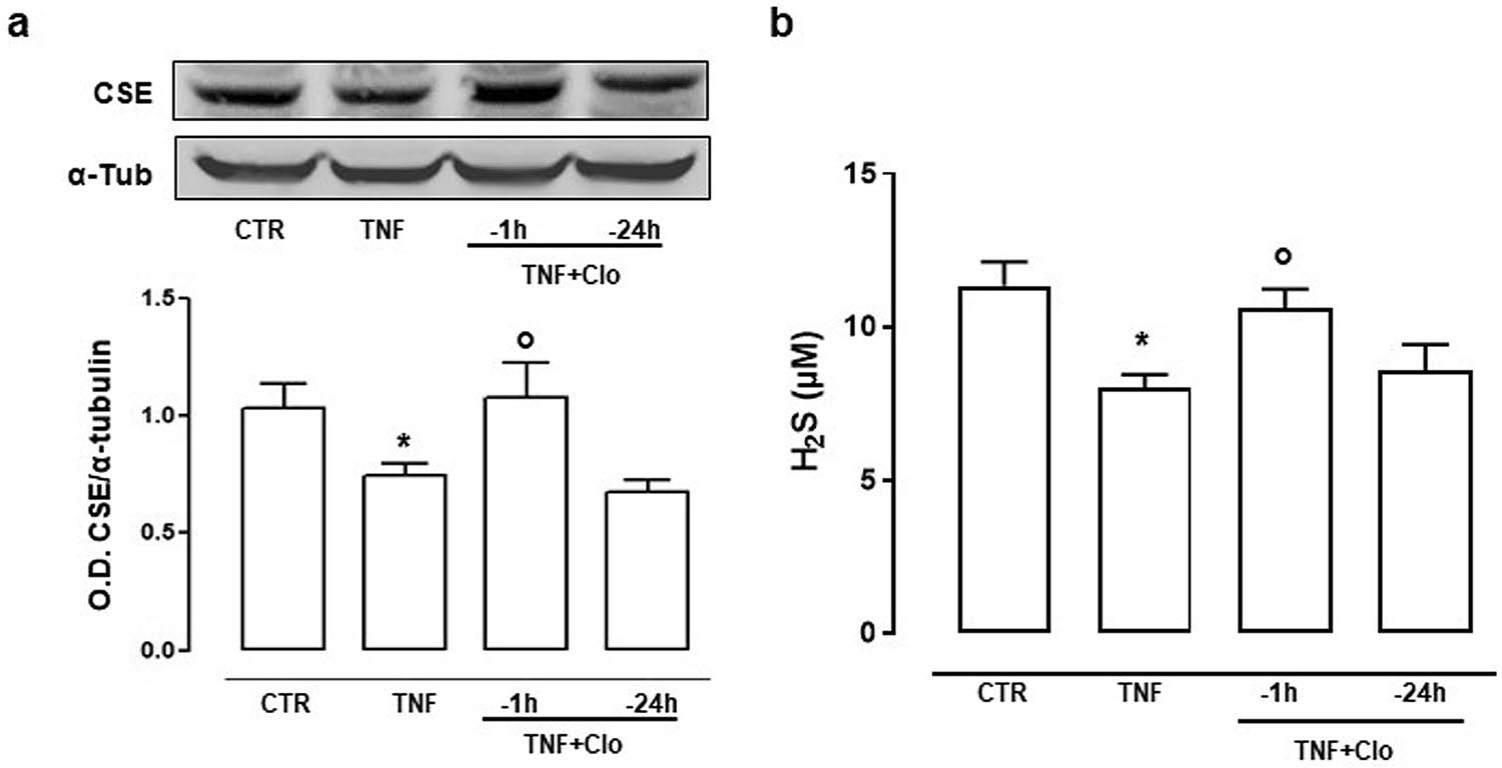
Time dependent effect by clodronate on CSE pathway following TNF-α stimulation in FLS. (**a**) Expression of CSE (cropped images) in FLS upon treatment with TNF-α alone or in combination with clodronate 1 h or 24 h before TNF-α stimulation. Optical density (OD) values have been standardized towards housekeeping protein α-tubulin (α-tub). *p < 0.05 vs CTR; °*p* < 0.05 vs TNF-α alone. (**b**) Extracellular H_2_S release following TNF-α alone or in combination with clodronate 1 h or 24 h before TNF-α stimulation. Data are expressed as concentration detected (µM). *p < 0.05 vs CTR; °*p* < 0.05 vs TNF-α alone. Full-length blots are presented in Supplementary Fig. 4.

We next tested whether the effect of clodronate was time-dependent and we performed experiments by using a short- or long-term exposure to clodronate. FLS were incubated with clodronate (10 µM) 24 h or 1 h before stimulation with TNF-α. Clodronate incubation 1 h before the inflammatory stimulus preserved cells by CSE suppression observed following TNF-α administration (Fig. 5a). Similarly, H_2_S levels in cell supernatants were equally preserved by clodronate treatment (Fig. 5b). Conversely, pre-treatment with clodronate 24 h before TNF-α challenge was ineffective (Fig. 5).

## Discussion

Clodronate is a non-nitrogen bisphosphonate used as anti-resorptive drug for both prevention and treatment of post-menopausal osteoporosis. Anti-fracture efficacy of oral clodronate has been demonstrated in several studies where the incidence of vertebral fractures at three years was 10.62% for group placebo versus 5.6% for the group treated with clodronate^25^. Interestingly, clodronate has been recently shown to generate pain relief with a good safety profile as well as to exert anti-inflammatory properties in patients^9^. However, there are no specific studies that have addressed the possible mechanism(s) underlying these effects.

Two key enzymes in inflammation are iNOS and COX_2_, which, under inflammatory conditions, generate high levels of nitric oxide (NO) and pro-inflammatory prostaglandins (PGs)^26^. In our study, the administration of clodronate, in presence of TNF-α, counteracted the increase in expression of iNOS triggered by TNF-α at concentrations as low as 0.1 µM, however no significant effect on COX_2_ expression was observed. This result indicates that the clodronate mechanism of action does not involve upstream inflammatory switches, such as NF-kB^27^, but interferes with specific pathways. Clodronate also modulated the cytokine release triggered by TNF-α.

It is well known that inflammatory mediators like interleukins drive the inflammatory response and lead to several phenomena, including pain^28–30^. Indeed, the pro-inflammatory cytokine pattern constituted by IL-1, IL-5, IL-6, IL-8 and IL-13 was significantly reduced by clodronate as well as it restored the expression of the anti-inflammatory cytokine IL-10. In addition, the expression of granulocytes specific factors such as GCSF, GMCSF and GRO was also suppressed by clodronate. Thus, clodronate seems to modulate the inflammatory process at different levels, in particular controlling the biosynthesis of factors responsible for leukocytes activation and differentiation, such as G-CSF, GM-CSF and GRO. This leads to reduction of cytokine release and, in turn, to suppression of the inflammatory pattern and associated pain^28–30^.

Notably, H_2_S donors promote analgesia based on activation and opening of K^+^_ATP_ channels^31^. It is known that drugs able to open these channels can induce anti-nociception by acting on central nervous system and this effect is associated to the release of endogenous opioids^32^. In addition, inflammatory response is associated to pain based on the release of pro-nociceptive cytokines such as TNF-α^33^. Thus, H_2_S seems to have a role in the regulation of the inflammatory pain, beyond its antinflammatory and pro-resolutive effect^23^.

Therefore, we further investigated whether H_2_S could be involved in the regulation of inflammatory cytokines responsible for pro-nociception during inflammation. H_2_S is known to modulate inflammatory response by limiting leukocyte trafficking and facilitating resolution of inflammation^23,34^. Thus, in our setting, we found that TNF-α administration to FLS reduced both H_2_S levels and CSE expression. This finding correlates with a pro-inflammatory profile possibly associated to onset of pain. Interestingly, we found that clodronate was effective in rescuing H_2_S biosynthesis, by modulating CSE expression as well as its activation. In particular, levels of H_2_S and CSE expression were restored following clodronate administration, taking levels back to those observed in control cells. This effect did not involve other H_2_S synthesizing enzymes CBS and 3MST, however, further experiments might be needed to better clarify whether CSE the only enzyme taking action in this mechanism. Nonetheless, we focused on CSE, as the only enzyme significantly modulated in its expression, and we also found that beneficial effect by clodronate was persistent following a short-term (1 h) pre-treatment with clodronate. This effect guarantees protection to cells by preventing the decrease of H_2_S levels observed following TNF-α challenge. On this basis, it is feasible that the effect of clodronate on pain relief could be dependent on its action in the control of H_2_S pathway, which limit the inflammatory response. This action is also time limited, as 24 h pre-treatment is not effective, thus indicating a short term action by clodronate in the control of H_2_S driven effects.

Indeed, our findings are in line with pain contrasting effects of clodronate found in the literature and that are not directly related to its known mechanism of anti-resorptive action. In particular, clodronate successfully treats complex regional pain syndrome in several patients^16^.

In another study, Frediani et al. tested the effect of intramuscular clodronate for symptomatic knee in OA patients. This study, enrolling 74 subjects, showed that administration of clodronate substantially decreased VAS score, improved function and reduced bone marrow oedema detected at MRI by WORMS scale^10^. Thus, clinical studies indicate the presence of an anti-inflammatory and analgesic efficacy that is also involved in preserving chondrogenesis^2^. This clinical evidence has been confirmed by using clodronate in different animal models^12,35^.

Interestingly, Suzuki et al. demonstrated that, in a mouse model of inflammation, zolendronate could enhance the increase of IL-1α and/or IL-1β induced by lypopolisaccharide (LPS). In addition, clodronate markedly abrogated the effect induced by zoledronate in presence of LPS, thus limiting its inflammatory process^36^.

In addition, Bonabello et al. and Kim et al. showed a significant pain reduction and anti-nociceptive effect exerted by clodronate, independently from its anti-resorptive effect^37–39^. It is to note that N-BPs causes exacerbation of joint inflammation, even though it showed a positive effect on structural bone damage, indicating that the anti-inflammatory and antinociceptive effects do not depend on a class effect^40^.

These evidences highlight that clodronate is able to control pain in several pathological conditions, although the underlying mechanisms have never been investigated. Here, we found that clodronate can modulate, in inflammatory setting, the generation of H_2_S. This effect could be linked to pain-relief action by clodronate, based on the fact that H_2_S can modulate targets of nociceptive response (K^+^_ATP_ channels) or stimuli triggered by inflammatory cytokines (TNF-α)^41,42^.

Similarly, the antinociceptive actions reported in the relevant literature for clodronate, could be also associated to the involvement of NO, together with H_2_S, as both gaseous mediators can per se modulate nociception^43^. In particular, NO has been described as a pro-nociceptive mediator, while H_2_S is able to suppress pain through the modulation of different channels (TRPV4, TRPA1, K^+^_ATP_)^44–46^. For the abovementioned reasons, we could suggest that clodronate may exert its anti-nociceptive effect through a dual mechanism involving the reduction in NO amount associated to the recovery of H_2_S levels. Nonetheless, this hypothesis needs further studies, analyzing in vivo models of nociception, to be verified.

**Figure 6:**
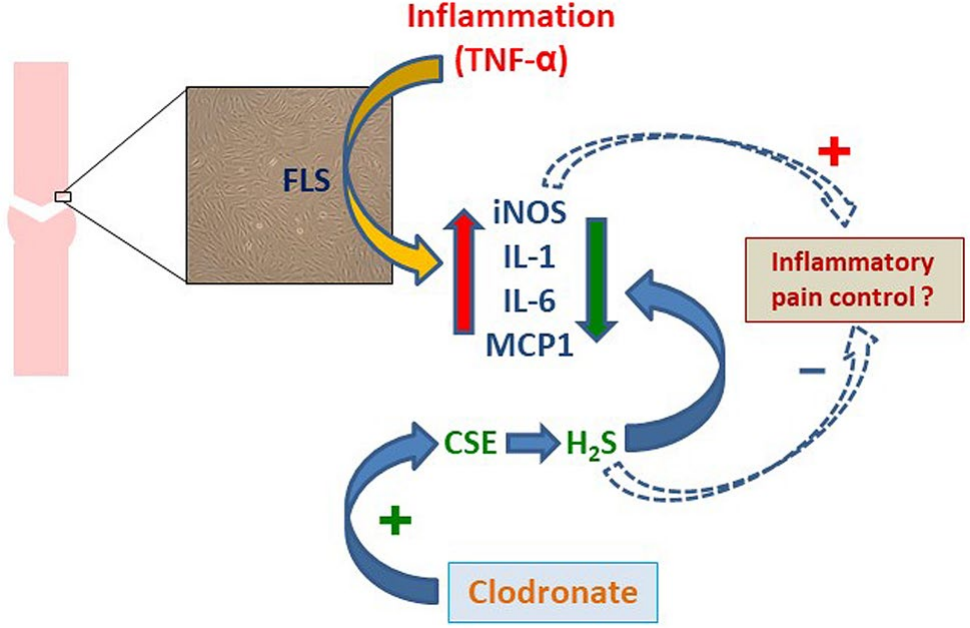
Schematic view of clodronate effect on TNF-α induced inflammation through modulation of H_2_S pathway.

Our in vitro study, though preliminary, highlights for the first time that the anti-inflammatory effects of clodronate reported in the literature might be associated to the modulation of H_2_S pathway in ongoing inflammation. Obviously, further investigation is needed as the underlying mechanism of the interplay between clodronate and H_2_S is far to be elucidated, also for a possible antinocipetive effect (Fig. 6). Nonetheless, the fact that clodronate is able to regulate levels of H_2_S (and NO) assure the control of the inflammatory state and provides a beneficial and peculiar effect, not known for other bisphosphonates. These findings may open new perspectives in the therapeutic scenario, where clodronate may acquire relevance, with particular attention to those diseases with a prominent inflammatory profile.

## Methods

### Cell culture

Immortalised non-aggressive fibroblast-like synoviocyte cell line (K4IM) were grown in T-25/T-75 flasks containing Iscove Modified Dulbecco’s Medium (DMEM), supplemented with 10% heat-inactivated FCS, 1% penicillamine/streptomycin and 1% L-glutamine. This cell line was established from primary fibroblast‐like synoviocyte cells by immortalization with SV40 T antigen^47^. Cells were cultured and splitted 1 to 5 every week and used starting from passage 4 to 6^48^.

### Experimental protocol

Cells were treated with Clo at different concentration (0.1–10 µM, 6–30 h) following administration of TNF-α (10 ng/ml, 6 h), used as inflammatory stimulus. The effect of Clo on K4IM was addressed by comparing the effects to vehicle treated cells undergoing TNF-α stimulation only. Experiments were repeated at least three times in duplicates.

### H_2_S levels determination

H_2_S levels have been determined by using a fluorometric assay based on SF7AM specific fluorochrome able to detect H_2_S^49,50^. Briefly, for what concerns cell supernatants, samples were diluted 1 to 4 in RIPA buffer and put in a 96-well black flat bottom plate. Then, SF7AM was added (10 µM) and plate was incubated a 37 °C under shaking conditions for 90 min to allow the fluorophore to quantitatively quench H_2_S and give an established fluorescence signal. In addition, H_2_S biosynthesis has also been measured, by using a different approach. Briefly, cell lysates obtained in RIPA buffer (0.5 mg/ml) have been incubated in 96-well black flat bottom and SF7AM was added (10 µM). Plate was incubated a 37 °C under shaking conditions for 90 min to allow for H_2_S biosynthesis and accumulation. In both cases, reading was performed (Ex 475 nm, Em 500–550) following incubation and H_2_S concentration was calculated against a Na_2_S standard curve (50 nM-200 µM).

### Western blot analysis

Cell pellets obtained from each experiment were mechanically homogenised in lysis buffer (RIPA supplemented with protease inhibitors cocktail) and total amount of protein was determined by using Bradford assay. Denatured proteins (60 µg) were separated on 10% sodium-dodecylsulfate polyacrylamide gels and transferred to polyvinylidene fluoride membrane (PVDF). Membranes were blocked in phosphate-buffered saline containing 0.1% v/v Tween-20 (PBST) and 3% w/v non-fat dry milk for 30 min, followed by overnight incubation at 4 °C with primary antibodies: mouse monoclonal anti-iNOS (1:500, BD-Pharmaingen), mouse monoclonal anti-COX-2 (1:2000, SantaCruz Biotechnologies), mouse monoclonal anti-CSE (1:1000, Proteintech), rabbit polyclonal anti-CBS (1:1000; Santa Cruz Biotechnologies), rabbit polyclonal anti-3MST (1:500, Novus Biologicals)^50^ or mouse monoclonal anti-tubulin (1:5000, Sigma-Aldrich). Membranes were extensively washed in PBST prior to incubation with horseradish-peroxidase conjugated secondary antibody for 2 h at room temperature. Following incubation, membranes were washed and chemiluminescence was detected by using ImageQuant-400 (GE-Healthcare). The target protein band intensity was normalised against housekeeping protein α-tubulin.

### Cytokine array panel

Cytokine determination was performed by using a commercially available kit (Abcam, UK) detecting 23 different cytokines. Array panel was developed according to manufacture indications. Cell samples from each treatment (control, CTR; TNF-α, TNF; TNF-α + clodronate, TNF + Clo) were homogenised and diluted to a final total protein concentration of 250 µg/ml. A separate membrane was used for each treatment. Membranes, following overnight incubation with samples at 4 °C, were washed and incubated with cocktail of biotin-conjugated anti-cytokine antibodies for 2 h at room temperature (RT), then washed again and incubated with HRP-conjugated streptavidin for 2 h at RT. Membranes were washed again and then chemiluminescence substrate was added. Dot spot signals were captured by using ChemiDoc Imaging System (BioRad).

### Statistical analysis

Optical density of western blot bands and array spots was determined by using imaging software ImageJ and data were expressed as mean ± SEM. For western blot analysis, relative OD was obtained by normalization against tubulin, taken as the reference protein. Analysis was performed by using ONE-way ANOVA, followed by Dunnett’s post hoc test and a *p* < 0.05 was taken as significant.

## Supplementary Information

Supplementary Information.

## Data Availability

The datasets generated during and/or analysed during the current study are available from the corresponding author on reasonable request.
