# Supplementary material for: In vitro evidence for the involvement of H2S pathway in the effect of clodronate during inflammatory response
Source: Sci Rep. 2021 Jul 20;11:14811. doi: 10.1038/s41598-021-94228-y (PMC8292495; doi:10.1038/s41598-021-94228-y)
Supplement: Supplementary file 1 — Supplementary Information. [file 41598_2021_94228_MOESM1_ESM.pdf]

Western blot images for antibody validation (data not shown)

CSE 42 kDa

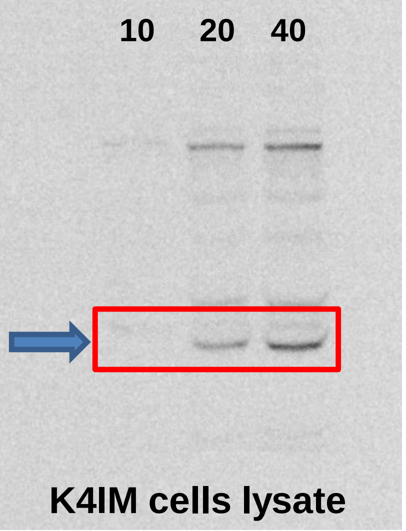

CBS 63 kDa

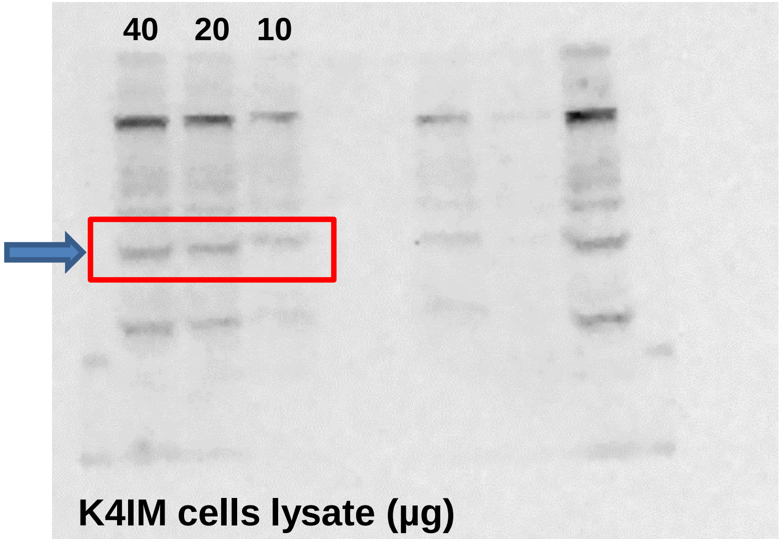

3MST 33 kDa

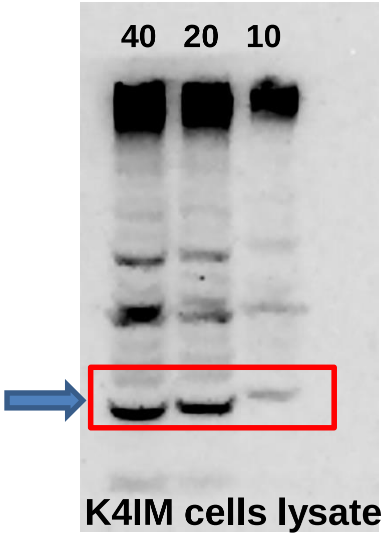

Supplementary figure 1

Full western blot acquisitions for cropped images in Figure 1

iNOS 130 kDa

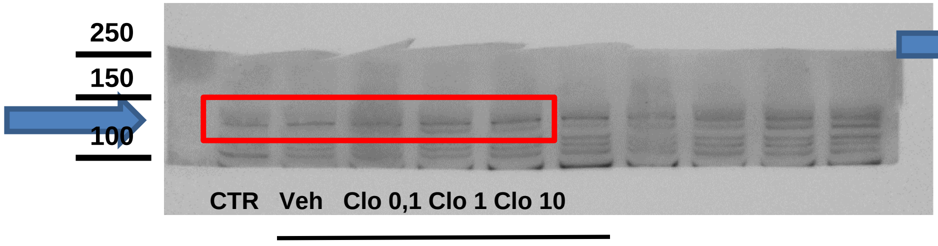

α-tubulin 50 kDa

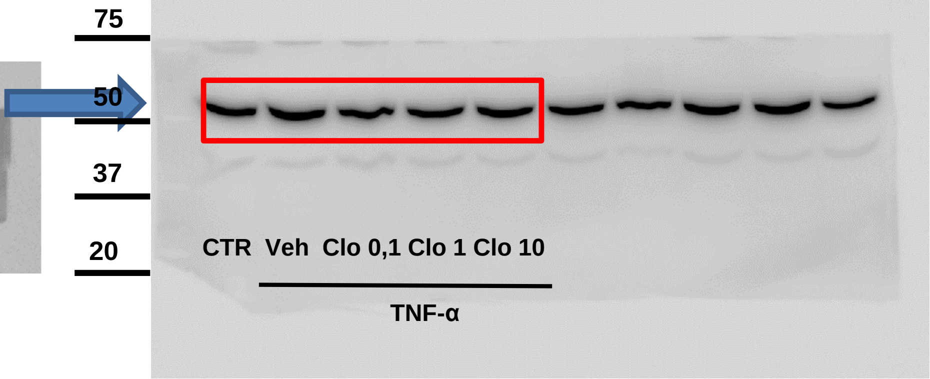

COX-2 70 kDa

α-tubulin 50 kDa

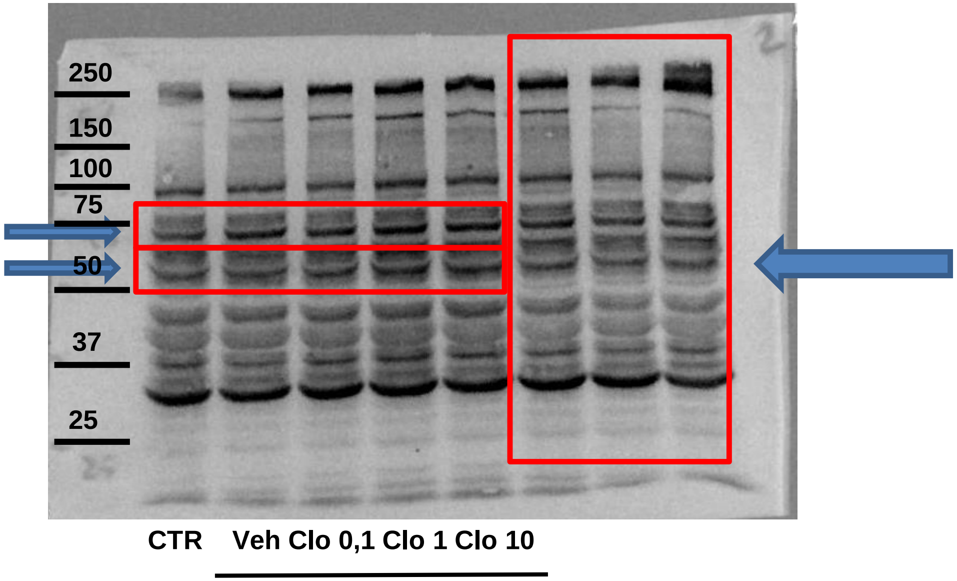

Same protocol applied to other compounds not relevant for the manuscript

Supplementary figure 2

Western blot images for cropped images in figure 4

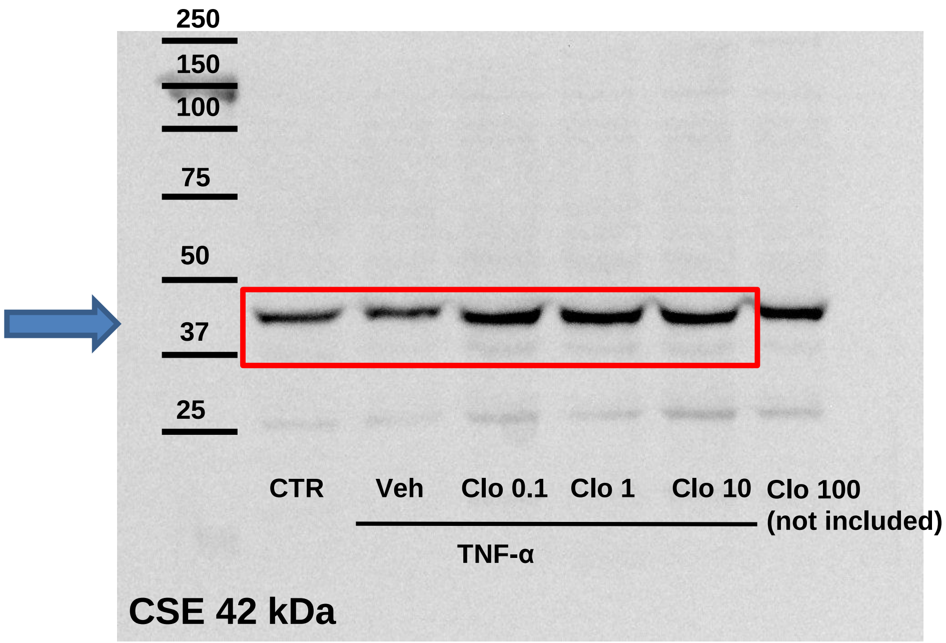

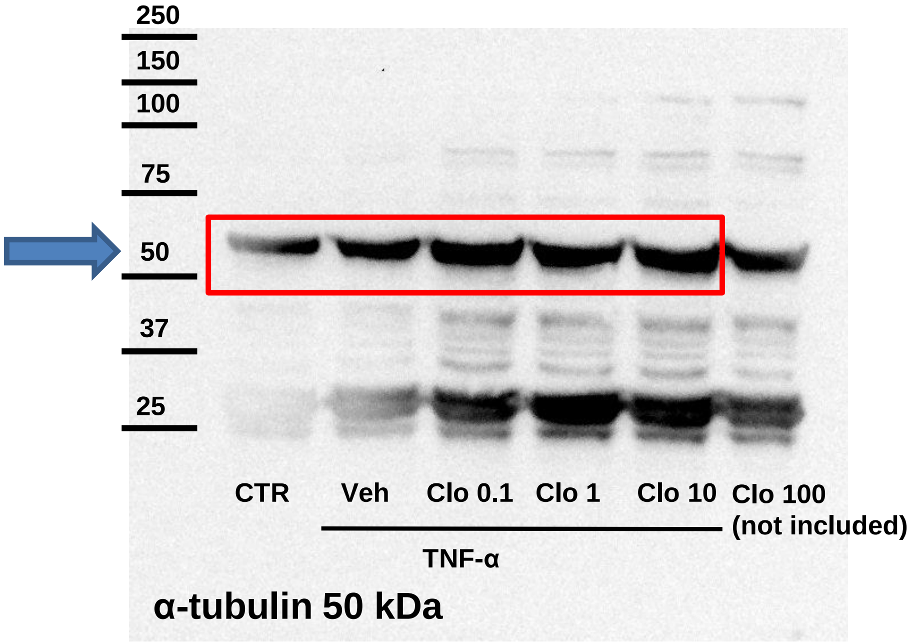

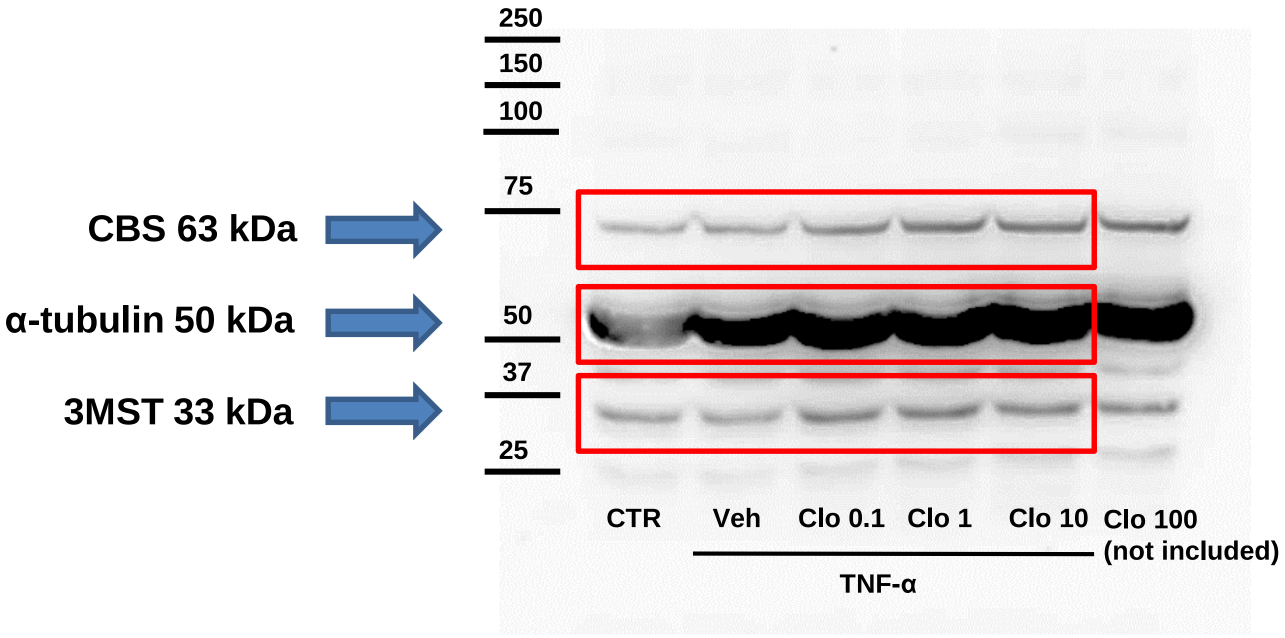

Supplementary figure 3

Western blot images for cropped images in figure 5

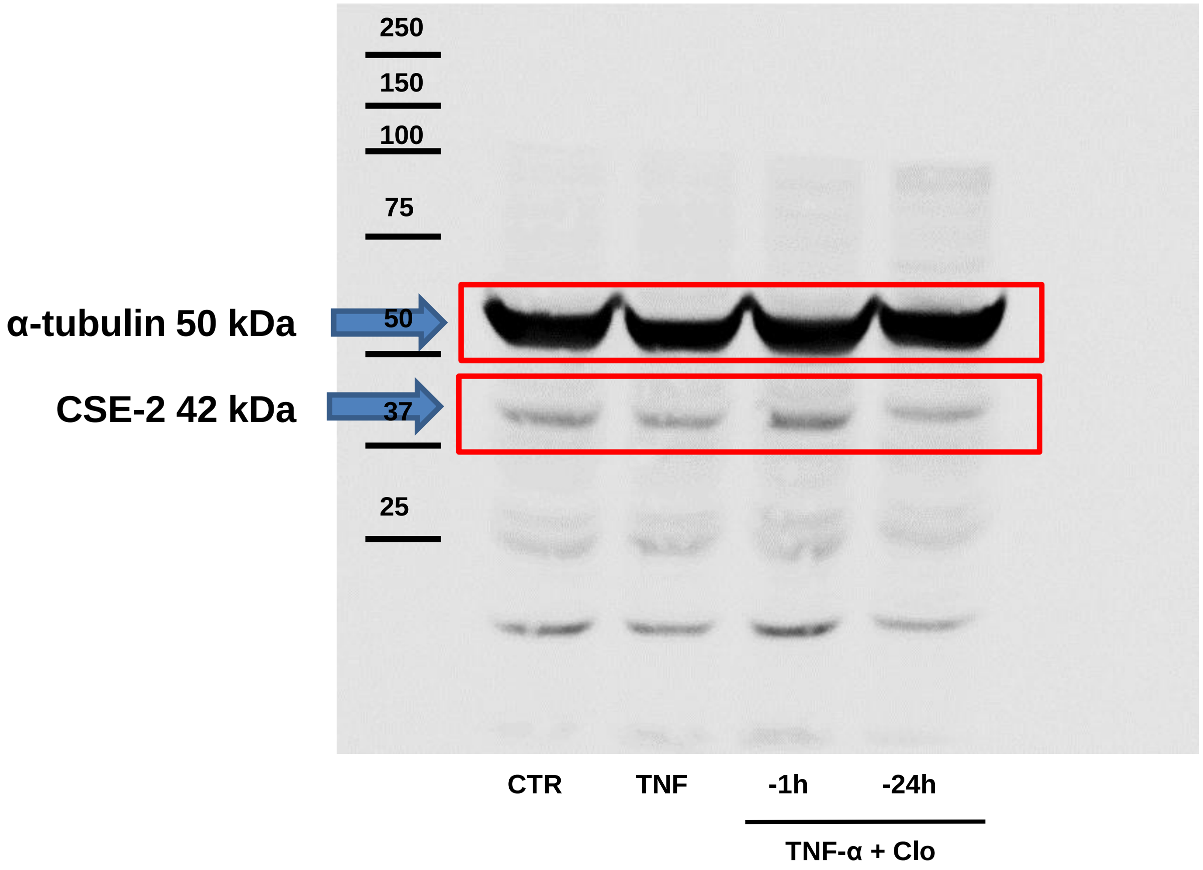

Supplementary figure 4
